# Biomarkers linking habitual short sleep duration with risk of cardiometabolic disease: current progress and future directions

**DOI:** 10.3389/frsle.2023.1293941

**Published:** 2023-12-05

**Authors:** Christopher M. Depner

**Affiliations:** Department of Health and Kinesiology, University of Utah, Salt Lake City, UT, United States

**Keywords:** sleep restriction, metabolomics, insufficient sleep, diabetes, cardiovascular disease, precision medicine, sleep health, sleep trackers

## Abstract

Approximately one in three adults in the United States sleeps less than the recommended 7 h per night. Decades of epidemiological data and data from experimental sleep restriction studies demonstrate short sleep duration is associated with adverse cardiometabolic risk, including risk of type 2 diabetes and cardiovascular disease. However, the precise mechanisms underlying this risk are not fully elucidated and there is a lack of sleep-based interventions designed to mitigate such risk. One strategy to overcome these limitations is to develop biomarkers that link habitual short sleep duration with adverse cardiometabolic risk. Such biomarkers could inform biochemical mechanisms, identify new targets for interventions, support precision medicine by identifying individuals most likely to benefit from sleep-based interventions, and ultimately lead to improved cardiometabolic health in people with habitual short sleep durations. Early progress demonstrates proof-of-principle that omics-based technologies are a viable approach to create biochemical signatures (biomarkers) of short sleep duration, primarily derived from acute studies of experimental sleep restriction. Yet, much work remains. Notably, studies that translate early findings from experimental sleep restriction to free-living adults with habitual short sleep duration have high potential to advance the field. Such studies also create an exciting opportunity for larger randomized controlled trials that simultaneously identify biomarkers of habitual short sleep duration and evaluate the efficacy of sleep-based interventions. Ultimately, early progress in developing molecular biomarkers of short sleep duration combined with the prior decades of progress in the sleep and metabolism fields provide the foundation for exciting progress in the biomarker development space.

## Introduction

In 2016, the Food and Drug Administration (FDA) and National Institutes of Health (NIH) convened a joint biomarker working group to address conflicting applications of the term “biomarker.” Especially pervasive issues were the conflicting standards, definitions, and intentions of how the term biomarker was being applied in clinical translational research. Such conflicting implementation of the term biomarker creates barriers to advancing research and to FDA review and approval of clinical biomarkers. The outcome of this FDA-NIH working group was the creation of the Biomarkers, EndpointS, and other Tools (BEST) Resource (FDA-NIH Biomarker Working Group, 2016), a living document designed for regular updates. As of September 12th, 2023, The BEST Resource defines a biomarker as a “characteristic that is measured as an indicator of normal biological processes, pathogenic processes, or biological responses to an exposure or intervention, including therapeutic interventions”, and indicates a biomarker may consist of molecular, histologic, radiographic, or physiologic characteristics (FDA-NIH Biomarker Working Group, 2016). Importantly, hard clinical outcomes including how a person feels, functions, or survives does not meet the BEST Resource definition of a biomarker (FDA-NIH Biomarker Working Group, 2016).

Coincidentally, along a similar timeline as the creation of the BEST Resource, the NIH and Sleep Research Society co-hosted a workshop entitled “Developing Biomarker Arrays Predicting Sleep and Circadian-Coupled Risks to Health”. A whitepaper report of this workshop highlighted a need to develop biomarkers that easily quantify daytime alertness and sleep and circadian health (Mullington et al., 2016). Given the adverse cardiometabolic consequences of poor sleep and circadian health (Depner et al., 2014), such biomarkers could theoretically have wide-reaching benefits including to help advance our understanding of sleep and circadian physiology, develop point-of-care diagnostics related to sleep and circadian disorders, predict risk of cardiometabolic disease linked to poor sleep and circadian health, track individual patient progress with sleep and circadian-based interventions, and predict which people may be most likely to experience improved health from sleep and circadian-based interventions (Mullington et al., 2016). The application of such biomarkers is generally consistent with the definition of a biomarker from the BEST Resource. Although such goals are appealing, we must also acknowledge the development of such biomarkers will be exceedingly challenging and time consuming, requiring years of complementary research from many teams. The long-term goal of such biomarkers should ultimately be to produce a healthier population with improved quality of life and lower mortality linked to short sleep duration (Mullington et al., 2016). In other words, such biomarkers are potential tools that could directly or indirectly help facilitate improved clinical outcomes including how a person feels, functions, or survives. The following *Perspective* article focuses on the risk of cardiometabolic disease linked to habitual short sleep duration and the potential benefits of developing biomarkers that represent or quantify such risk. Although I focus on sleep duration, the concepts discussed are easily applied to other dimensions of sleep health such as regularity, satisfactions, alertness, timing, and efficiency (Buysse, 2014).

## Needs and potential significance of biomarkers that quantify risk of cardiometabolic disease associated with short sleep duration

The American Academy of Sleep Medicine and the Sleep Research Society recommend adults regularly obtain at least 7 h of sleep per night to promote optimal health (Watson et al., 2015a,b). Despite these recommendations, over one in three adults in the United States report sleeping <7 h per night, obtaining habitual short sleep duration (Centers for Disease Control and Prevention, 2014; Ford et al., 2015). Such habitual short sleep duration is consistently associated with higher risk of cardiometabolic disease including obesity, diabetes, and cardiovascular disease, the leading cause of death in the United States (Cappuccio et al., 2008, 2010, 2011; Knutson et al., 2009, 2011; Liu et al., 2013; Depner et al., 2014; St-Onge et al., 2016). Additionally, in 2022 the American Heart Association added “healthy sleep” as the 8th component to Life's Essential 8 for lifelong good health (Lloyd-Jones et al., 2022). Furthermore, recent data show that adding a sleep outcome on top of traditional cardiovascular disease risk factors creates stronger associations with incidence of cardiovascular disease compared to the traditional risk factors without considering sleep (Makarem et al., 2022). These findings further indicate poor sleep health is a potential independent risk factor for cardiometabolic disease. Data from laboratory trials of experimental sleep restriction further contribute to the evidence linking short sleep duration with risk of cardiometabolic disease. Specifically, data consistently shows experimental sleep restriction causes adverse cardiometabolic risk including impaired insulin sensitivity, increased food intake, later timing of food intake, positive energy balance, weight-gain, and increased blood pressure (Spiegel et al., 1999; Broussard et al., 2012; St-Onge et al., 2012; Depner et al., 2014; Eckel et al., 2015; McNeil and St-Onge, 2017; Makarem et al., 2019). Despite the body of evidence linking short sleep duration with adverse cardiometabolic risk, there are currently no established biomarkers, molecular or otherwise, that convincingly represent or quantify such risk among the general population. In the long-term, developing such biomarkers could potentially help mitigate the morbidity and mortality of cardiometabolic disease linked to short sleep duration.

Multiple platforms could be implemented to develop such biomarkers. Some examples are (1) omics-based technologies for molecular biomarkers, including metabolomics, proteomics, and transcriptomics analyses that can quantify thousands of metabolites, proteins, or genes in a single sample. These omics-technologies are commonly applied to blood samples but can also be applied to tissues and other bodily fluids. Additionally, microbiome analyses of stool samples fit within this category of omics-based technologies. (2) Wearable technology that quantifies physiological parameters like interstitial glucose, heart rate, skin temperature, or sleep itself [e.g., wrist-actigraphy/consumer wearables (Depner et al., 2019)]. (3) Polysomnography (PSG), the current gold-standard for quantifying sleep (Rechtschaffen, 1968). A biomarker could have different end-uses pending the platform used. For example, molecular biomarkers could help inform biochemical mechanisms and potentially serve as point-of-care biomarkers for clinicians to identify individuals with elevated disease risk. Wearable devices could help consumers, patients, and clinicians potentially identify risk and longitudinally track risk profiles. PSG could potentially help identify specific stages or features of sleep that are most strongly linked to cardiometabolic disease risk. All these use cases could potentially help inform new sleep-based interventions with the goal of improving sleep to improve cardiometabolic health. Despite the theoretical promise of such biomarkers, to date, no biomarkers exist that truly fulfill the end goal of creating a healthier population with improved quality of life and lower overall mortality linked to short sleep duration. Thus, there is still a large gap in knowledge the field must overcome to realize these potential long-term benefits.

## Sleep biomarkers: current data and limitations

As of September 12th, 2023, a PubMed (https://pubmed.ncbi.nlm.nih.gov/) search for “*sleep” AND “biomarker”* returned 2,184 publications (Table 1). This search illustrates an expanding effort is being invested in the sleep biomarker space with 86 publications in 2013, 367 publications in 2022, and 321 publications to date in 2023. Thus, manuscripts published since 2022 account for 32% of the total. I focus on molecular biomarkers but the broad principles and needs for robust biomarker development apply across biomarker platforms.

**Table 1:** Representative example biomarkers of sleep duration.

**Biomarker platform**	**Change with short sleep duration**	**Strengths**	**Key References**
**Wearable and clinical platforms**			
Wrist-actigraphy	Decrease	•Longitudinal data •Ease of use	Ancoli-Israel et al., 2015
Commercial sleep trackers	Decrease	•Longitudinal data •Real-time data	Chinoy et al., 2020
Polysomnography	Decrease	•Gold-standard •EEG analysis •Clinical sleep disorder diagnostics	Rechtschaffen, 1968
**Biomarkers derived from studies of experimental sleep restriction**			
Amylase activity and gene expression	Increase with sleep deprivation in flies and humans	•Saliva is easily sampled •Consistent data in flies & humans	Seugnet et al., 2006
Untargeted and targeted blood metabolomics	100s of increased and decreased metabolites in rats and humans	•High throughput •Biochemical insights •Captures broad molecular changes	Depner et al., 2020 Weljie et al., 2015 Davies et al., 2014
Urine metabolomics	Eight increased and 8 decreased metabolites	•High throughput •Urine is easily sampled •Captures broad molecular changes	Giskeodegard et al., 2015
Blood Transcriptomics (gene expression)	Many altered “signatures” consisting of 9–74 genes	•High throughput •Better performance to quantify acute chronic sleep restriction •Show associations with impaired cognition during total sleep deprivation	Laing et al., 2019 Uyhelji et al., 2018
**Biomarkers derived from cross-sectional or longitudinal data from “free-living” environment**			
Blood metabolomics	Metabolomics signatures of 9–153 metabolites	•High throughput •Show associations with elevated risk of coronary heart disease and diabetes •Derived from larger sample sizes with higher statistical power	Zhuang et al., 2023 Huang et al., 2019

Table 1 summarizes platforms and strengths of biomarkers of sleep duration. In 2006, salivary amylase activity and gene expression were identified as potential biomarkers of sleep drive in flies and humans (Seugnet et al., 2006). Cross-sectional data have also identified higher salivary amylase in people with low vs. high sleep efficiency, although there were no differences in salivary amylase in this same dataset between people with less than vs. more than 6 h of sleep per night. Although these data show promise, it is not established if changes in salivary amylase due to poor or disrupted sleep could potentially identify or represent adverse cardiometabolic risk linked to habitual short sleep duration. More recently, several groups have used metabolomics and transcriptomics in studies of experimental sleep restriction to identify potential molecular signatures of acute short sleep duration or total sleep deprivation (Bell et al., 2013; Davies et al., 2014; Chua et al., 2015; Weljie et al., 2015; Uyhelji et al., 2018; Laing et al., 2019; Depner et al., 2020). In these studies, a molecular signature refers to the concept that a single biomarker comprises alterations in the expression or levels of multiple genes or metabolites. This approach holds promise for capturing changes in biochemistry across multiple tissues and biochemical pathways. This is especially appealing for developing sleep biomarkers because short sleep duration has a wide spectrum of adverse physiological health consequences. Although most work to date is derived from blood samples, other biological matrices such as urine, saliva, sweat, and breath condensate are viable considerations (Giskeodegard et al., 2015; Scholz et al., 2023). In general, the current data show proof-of-principle that acutely (e.g., 2–7 days) restricting sleep duration in otherwise healthy young adults, that habitually sleep at least 7 h per night, alters abundance of gene expression and metabolites. In the current evidence no consistent findings have emerged to suggest biomarkers based on metabolites or gene expression will produce superior biomarker performance. The existing data do however support the hypothesis that a biomarker signature (collection of changes in expression or levels of multiple genes or metabolites) of habitual short sleep duration in free-living adults could be developed. Furthermore, findings from most of these studies suggest sleep restriction alters lipid metabolism. Notably, changes in phospholipids, diacylglycerols, and sphingolipids have been reported across studies (Bell et al., 2013; Weljie et al., 2015; Depner et al., 2020), strengthening the evidence that sleep restriction may adversely impact these classes of lipids. Although the lipid classes impacted by sleep restriction are consistent, it is worth noting these lipid classes represent 1,000s of individual lipids and thus specific lipids show little overlap between studies to date. Additionally, most metabolomics platforms detect many more lipids than non-lipid compounds which could bias results toward detecting changes in lipids. Targeted metabolomics analyses across many studies will be required to overcome these limitations. Regardless of limitations, many of the reported lipids impacted by sleep restriction are linked with risk of diabetes and cardiovascular disease (Funai et al., 2020; Tippetts et al., 2021). This raises the exciting hypothesis that short sleep duration impacts these lipid pathways in a manner that increases cardiometabolic risk. This hypothesis warrants more detailed investigation, especially in at-risk populations such as older adults with obesity.

Changes in many blood metabolites have been linked with sleep duration and other aspects of sleep health in cross-sectionals and longitudinal studies. In some studies, altered levels of metabolites were associated with risk of diabetes and CVD in people with short or disrupted sleep compared to people with adequate sleep (Huang et al., 2019; Zhuang et al., 2023), but in other studies no such associations were detected (Fritz et al., 2023). Where significant associations were detected, the metabolites included many lipids that also changed in studies of experimental sleep restriction, including diacylglycerols, phospholipids, lysophospholipids, and sphingolipids (Bell et al., 2013; Weljie et al., 2015; Depner et al., 2020). These analyses should be considered secondary or exploratory as the studies were not explicitly designed to identify metabolite-based biomarkers of sleep that are linked with adverse cardiometabolic risk, and therefore findings are truly hypothesis generating. Furthermore, prior work shows up to 33% of the human plasma metabolome is regulated by the circadian system, and it is established that short sleep duration leads to circadian misalignment (Dallmann et al., 2012; Chua et al., 2013; Eckel et al., 2015; Skene et al., 2018). Future work is needed to precisely disentangle the potential impacts of short sleep duration vs. circadian misalignment on metabolomics-based biomarkers. This is an important step as this knowledge could help better tailor interventions to sleep or circadian focused changes in health behaviors.

The field has made progress in identifying potential molecular biomarkers of short sleep duration, but there are limitations that must be addressed. First, many different metabolomics platforms, including targeted and untargeted approaches, were used to generate data from different studies. These varying metabolomics methods impede directly comparing data between studies which creates a major barrier to replicating findings and conducting biomarker performance analyses in completely independent studies. As biomarker efforts progress it will be critical to use untargeted omics data as hypothesis generating to inform specific targeted assays. Additionally, for promising biomarkers, it is imperative to show the data replicate between studies and across labs using standard analytical techniques. Second, many of these published findings are considered secondary or exploratory, are underpowered to draw strong conclusions from the high-dimensional omics-based data, and therefore are not optimally designed for developing biomarkers of habitual short sleep duration. Third, data from studies of experimental sleep restriction are short-term and derived from otherwise healthy adults that habitually obtain at least 7 h of sleep per night. Furthermore, these laboratory studies rigidly control food intake and sleep timing such that participants receive exactly 4 or 5 h of time in bed per night with bed and waketimes controlled to the exact second. Such rigid control is a strength and a limitation as it helps eliminate potential confounding but also decreases generalizability. As such, it is unclear if data from these laboratory studies translate to people with habitual short sleep duration outside the laboratory in the “free-living” environment. Critically, to develop biomarkers that link risk of cardiometabolic disease with habitual short sleep duration, such biomarkers must be developed and tested in the relevant/target populations (i.e., people with habitual short sleep duration and elevated cardiometabolic risk) which will necessitate much larger samples sizes. Fourth, although the existing data has linked potential biomarkers with impaired cognitive performance (Uyhelji et al., 2018) and changes in levels of toxic lipids (Bell et al., 2013; Weljie et al., 2015; Depner et al., 2020), no such biomarkers have convincingly linked habitual short sleep duration with incidence of cardiometabolic disease. Fifth, it is currently unknown how any sleep-based interventions may influence the currently identified set of potential biomarkers. Taking this one step further, it is also unknown if an intervention that could “improve” such biomarkers ultimately leads to improved cardiometabolic health. As already noted, the current data create a platform to conduct more rigorous studies designed to develop biomarkers that link habitual short sleep duration with adverse cardiometabolic risk.

## Future research to advance the field

### Biomarker performance quantification

Recommendations for wearable device data in the sleep field (e.g., actigraphy and commercial wearables) have called to use the term “performance” instead of “validation” because there is no single threshold for validation, especially across populations (Goldstein and Depner, 2021; Menghini et al., 2021). A similar framework is warranted in molecular, or other, biomarkers that link habitual short sleep duration with risk of cardiometabolic disease. Robust biomarker performance quantification studies are critical to ensure the biomarker is clinically relevant. Even if a potential molecular biomarker is not being developed for use in clinical settings, but perhaps to identify biochemical mechanisms, it is critical to ensure the biomarker translates to the target population of interest. Regardless of the end goal, all biomarker performance evaluation studies must be conducted in cohorts that are completely independent from the discovery dataset and optimally will be conducted across multiple labs and multiple cohorts. Beyond accuracy, other performance metrics to consider are sensitivity, specificity, positive predictive value, negative predictive value, area under the receiver operator characteristic curve, and number needed to screen when implementing for clinical use. Related, for development and performance evaluation studies it is important to consider how sleep itself is being quantified and over what duration. Given the limitations of long-term PSG over weeks to months, a viable approach is wrist-actigraphy or commercial sleep trackers. Although beyond the scope of the current discussion, it is worth noting that many commercial sleep tracking devices use black-box technology and algorithms that are frequently updated and could potentially impact the calculation of sleep duration in biomarker studies. Recommendations for use of these approaches exist elsewhere and it is essential to adhere to best practice recommendations (Ancoli-Israel et al., 2015; Depner et al., 2019; Menghini et al., 2021).

### Clinical implementation

Questionnaires to assess sleep duration, sleepiness, alertness, and other dimensions of sleep health already exist, such as the Pittsburgh Sleep Quality Index (Buysse et al., 1989), and are relatively simple to administer in clinical settings. Optimally, any new biomarkers will have a relatively similar burden for the patient and clinician and identify habitual short sleep duration with superior performance compared to these existing self-report options. In other words, a new biomarker needs to help us more precisely identify people with habitual short sleep duration compared to without the biomarker. If this target is achieved and the biomarker improves our capacity to identify people who are at elevated risk of cardiometabolic disease due to their habitual short sleep duration, we must also develop interventions that mitigate such risk. Otherwise, simply informing someone they are at elevated risk of disease due to their habitual short sleep duration, without providing an actionable intervention, has the risk of creating anxiety, worry, and potential medical-care seeking behavior that could elicit more severe adverse side-effects than simply doing nothing at all. Simply stated, a biomarker on its own is not an intervention. However, in the initial stages, developing molecular biomarkers without actionable interventions is a worthy endeavor as such biomarkers could inform novel interventions. A consideration for future studies is to focus on biomarker and intervention development in parallel. For example, it may be possible to test sleep-based interventions in randomized controlled trials that are powered for assessing potential biomarkers and clinical endpoints like major adverse cardiac events or incidence of diabetes.

### Timing and dose

We need to identify the optimal age range (timing) where we can expect a patient to have a high likelihood of benefit from a sleep-based intervention. We also need to understand how the dose, in magnitude and duration (weeks/months/years/decades), of habitual short sleep duration influences clinical outcomes from the perspectives of risk profiles and potential benefits from interventions. Most importantly, we need to know when it is possible to change an individual patient's trajectory toward health or disease based on the feedback and action informed by biomarkers.

### Ethical considerations

Short sleep duration is linked with many adverse health outcomes including risk of obesity, diabetes, cardiovascular disease, Alzheimer's disease, and impaired cognition (Depner et al., 2014; Hudson et al., 2020; Mander, 2020). These health consequences have the potential to increase health care costs and carry deadly public health consequences, especially in safety critical industries like the medical, transportation, and military fields. As such, there are important considerations regarding clinical biomarkers of habitual short sleep duration in electronic health records. A key consideration is determining and controlling who has access to such data. For example, such a biomarker could have implications for determining pre-existing conditions for health insurance, job hiring, accident investigations, and liability of health care costs, just to name a few. Beyond molecular biomarkers of sleep duration, these ethical considerations are likely to become increasingly relevant in modern society and warrant dedicated research as the field progresses.

## Conclusion

Over the prior decades the sleep field has made incredible progress identifying habitual short sleep duration as a risk factor for cardiometabolic disease. This work is in part highlighted by the American Heart Association recognizing “healthy sleep” as one of Life's Essential 8 for lifelong good health (Lloyd-Jones et al., 2022). Despite this progress there is a lack of established sleep-based interventions designed to improve cardiometabolic health in people with habitual short sleep duration. Developing biomarkers that link habitual short sleep duration with adverse cardiometabolic risk could help inform such interventions and advance the field. Early data in this field show proof-of-principle that developing such biomarkers is possible but much work remains. Notably, there is a large need to design studies that translate these early findings from laboratory studies of experimental sleep restriction to free-living adults with habitual short sleep duration. This represents an exciting opportunity to conduct larger randomized controlled trials that are powered to simultaneously identify biomarkers of habitual short sleep duration and evaluate the impact of sleep-based interventions on cardiometabolic health outcomes. Such rigorously controlled randomized trials will be critical to continue the biomarker development pipeline and to ultimately help improve overall quality of life for people with habitual short sleep duration. Developing biomarkers that link habitual short sleep duration with adverse cardiometabolic risk holds great potential and the field is poised for ongoing progress in the coming decade.

## Data availability statement

The original contributions presented in the study are included in the article/supplementary material, further inquiries can be directed to the corresponding author.

## Author contributions

CD: Funding acquisition, Writing – original draft, Writing – review & editing.
